# Health economists, tobacco control and international development: On the economisation of global health beyond neoliberal structural adjustment policies

**DOI:** 10.1057/biosoc.2013.3

**Published:** 2013-04-22

**Authors:** David Reubi

**Affiliations:** a School of Geography, Queen Mary, University of London, Mile End Road, London E1 4NS, UK. E-mail: d.reubi@qmul.ac.uk

**Keywords:** global health, (health) economics, neo-liberalism, structural adjustment, taxation, tobacco control

## Abstract

This article addresses the increasing influence of economic rationalities in global health over the past 30 years by examining the genealogy of one economic strategy – taxation – that has become central to international anti-smoking initiatives in the global South. It argues that this genealogy sits uncomfortably with the usual story about economics and global health, which reduces the economisation of international health to neoliberal structural adjustment policies aimed at stabilisation, liberalisation and privatisation and laments their detrimental effect on health. While not disputing these policies' importance and damaging impact, the genealogy of tobacco taxes outlined in this article shows that the economisation of global health is not only about neoliberal structural adjustment policies but also about sin taxes, market failures and health economics. By stressing how changes in health like the global South's epidemiological transition can impact on economics and how beneficial taxation can be for health, it also shows that the relation between economics and health is not always unidirectional and detrimental to the latter. In doing so, the article contributes to the critique of the often mechanical use of neo-liberalism to explicate change and calls for other stories about the economisation of global health to be told.

## Introduction

Over the past 30 years, economic rationalities have played an increasingly significant role in international efforts to promote health in the developing world (Thomas and Weber, 2004; Brown *et al*, 2006). Perhaps the most striking symbol of this transformation is the way the World Bank – an institution where economists and economic rationalities dominate – has progressively come to replace the World Health Organization (WHO) as the most important actor in the field of international health (Abbasi, 1999). But there are many other signs of economics' growing influence on global health. Graduates in economics, for example, have become a familiar presence among organisations active in the field of health in the global South. There are also a growing number of books, articles and reports that examine different economic aspects of health care and health services in developing countries. Similarly, economic concepts like ‘efficiency' and ‘price elasticity' as well as economic techniques like cost-benefit analyses and demand curbs modelling inform an ever-rising number of international health initiatives funded by philanthropies and development agencies.

The present article addresses this economisation of global health through the examination of one economic strategy – taxation – that has become central to most existing international initiatives purporting to curb tobacco consumption in developing countries. Taking a genealogical approach (Rabinow, 1989; Foucault, 2004; Reubi, 2010), the article identifies and traces the articulation of the intellectual concepts, expert networks, political theories and techniques that have made taxation so critical in global tobacco control today. Specifically, it argues that three developments in particular have made it possible for taxation to become central to current anti-smoking policies and interventions in the global South: (1) the elaboration of a new body of knowledge on tobacco taxes by North American and European health economists from the early 1970s onwards; (2) the problematisation of the tobacco epidemic and, more broadly, non-communicable diseases (NCDs) in developing countries from the late 1970s onwards; and (3) the active promotion of tobacco taxes by a network of epidemiologists and economists centred around the World Bank at the turn of the twenty-first century.

As the article further shows, this genealogy of tobacco taxes sits uncomfortably with the usual story about economics and global health found in the literature (for example, Whitehead *et al*, 2001; Thomas and Weber, 2004; Rowden, 2009). This story equates the economisation of global health with the structural adjustment policies aimed at stabilisation, liberalisation and privatisation that became dominant in international development in the early 1980s. These policies, the story goes on, are the product of a longstanding neoliberal critique of development economics and have, through the way they transformed the organisation and financing of health care, been extremely detrimental to health in the global South. The genealogy presented in this article does not dispute that neo-liberal structural adjustment policies have played a critical and often damaging role in international health over the past 30 years. But, contributing to a growing literature that questions the way neo-liberalism is mechanically used to make sense of change (for example, Ferguson, 2009; Collier, 2011), it challenges some of the limitations inherent to the standard story about economics and global health. In particular, it shows that the economisation of global health is not only about the neoliberal critique of development economics and structural adjustment policies but also about health economics, rational addiction theory, market failures and sin taxes. Furthermore, by stressing both how changes in health like the global South's epidemiological transition have influenced economics and how beneficial tobacco taxation can be for health, it also shows that the relation between economics and health is not always unidirectional and detrimental to the latter.

Before tracing the genealogy of tobacco taxes and showing how it jars with the standard story on the economisation of global health, the article describes the key role that taxation plays in international efforts to curb tobacco consumption in developing countries today.

## Taxation and Global Tobacco Control

Over the past 10 years, taxation has become the most important tool in international efforts to curb tobacco consumption in the global South. As the WHO (2009, p. 56) explains in one of its latest *Annual Report on the Global Tobacco Epidemic*:

Increasing the price of tobacco products through significant tax increases is the single most effective way to decrease tobacco use.

Similarly, the Framework Convention Alliance (2012) – probably the world's largest anti-smoking advocacy group today – argues that:

Increasing the price of tobacco products [through taxation] is one of the most effective tobacco control measures available.

The important role played by taxation is also visible in the amount of financial support it receives from international tobacco control initiatives run by philanthropies and development agencies located in the global North. For example, the Canadian International Development and Research Council (IDRC) – one of the first development agencies to fund programmes on tobacco control in the global South through its *Research on International Tobacco Control Initiative* – has regularly funded projects on taxation in countries like South Africa, China, Mexico and Tanzania (IDRC, 2012). Another illustration is the Rockefeller Foundation's 2000–2004 *Trading Tobacco for Health Initiative,* the first substantial grant for global tobacco control to come from the philanthropic sector, which funded a team of American economists led by Frank Chaloupka to develop effective tobacco taxation policies in South-East Asia (Rockefeller Foundation, 2001, p. 84; Rockefeller Foundation, 2003, p. 47). Similarly, the *International Tobacco and Health Research and Capacity Building Program* run by the National Institutes of Health's Fogarty International Centre has financed American economists like Tei-Wei Hu to build a knowledge base for tobacco tax policies in Indonesia and China (Fogarty International Centre, 2012). More recently, both Bloomberg Philanthropies and the Gates Foundation have identified taxation as a priority among the interventions to curb the smoking epidemic in the developing world for which they give financial support (Frieden and Bloomberg, 2007, p. 1759; Gates Foundation, 2009, p. 1).

A further sign of the importance of taxation for global tobacco control today is the large body of knowledge on tobacco taxes in developing countries contained in public health textbooks, health economics journals, reports to funders and manuals. A key element in this body of knowledge is the medical, political and philosophical arguments in favour of using taxation in the global South. These arguments comprise: the possibility to reduce the high mortality rates associated with smoking; the ability to decrease the medical costs and lost days of productivity due to tobacco consumption; and the fact that the tobacco market does not work because of information failure and negative externalities (for example, Guindon *et al*, 2003; Ross and Chaloupka, 2006; Chaloupka *et al*, 2011). Another important element in this body of knowledge is the models, methods and techniques to set up a taxation system, including: tax models; methods to determine efficient tax rates; econometric procedures to forecast tax revenues; physical controls and stamps; systems of registration and licensing; and techniques to calculate compliance rates (for example, Yurekli and De Beyer, 2004; Van Walbeck, 2010; WHO, 2010; International Agency for Research on Cancer, 2011). A further, critical element in this body of knowledge is the analyses of existing tobacco tax policies in developing countries like China, India, Mexico and Russia. They comprise: an outline of the national tobacco market, from the regulatory environment and the structure of the tobacco industry to the consumers' income levels, values and tastes; a description of the country's tax policy; and an estimate of the price elasticity of cigarettes (for example, Sarntisart, 2003; Ross and Przewozniak, 2004; Tsai *et al*, 2005; Jimenez-Ruiz *et al*, 2008; John *et al*, 2010).

Another, final indication of the vital role taxes play in international tobacco control today is the increasing number of economics graduates with some competence in econometric analysis working on taxation. One can find them not only in economics departments and schools of public health in the global North but also, thanks to the capacity building efforts of the IDRC, the Rockefeller Foundation and the Fogarty International Centre, in the global South. One can also increasingly find them working directly for organisations active in international tobacco control. One example is the Tobacco Free Initiative (TFI), the WHO unit in charge of tobacco control, which spearheaded the negotiations that lead to the adoption of the *Framework Convention on Tobacco Control* (FCTC), and one of the five current key partners of the *Bloomberg Initiative to Reduce Tobacco Use* (BI). Since the mid 2000s, TFI has employed a team of economists led by Aida Yurekli to offer technical support on tobacco taxation to WHO member states. This technical support has included running workshops for the personnel of finance ministries and the publication of the 2010 *WHO Technical Manual on Tobacco Tax Administration*. Another example is the American Cancer Society (ACS) – an NGO that has long been key to international efforts to curb the tobacco epidemic in the global South. A few years ago, ACS hired an international team of economists directed by Hana Ross. An important part of their work has been to analyse tobacco tax policies in different developing countries for the BI and to offer technical support to the *Southeast Asian Tobacco Tax Initiative* funded by the Gates Foundation.

## The Economics of Tobacco and Taxation

The first development that was critical in making taxation an essential tool of current international anti-smoking efforts is the elaboration of a new body of knowledge on tobacco taxes by North American and European health economists from the 1970s onwards. As this section shows, this knowledge transformed the way taxation was conceptualised among public health and tobacco control experts.

The field of health economics emerged and progressively established itself between the 1960s and the 1980s, first in the USA and the United Kingdom and later in other European countries (Colvin, 1985, chapter 1; Fuchs 1987; Ashmore *et al*, 1989, chapter 1; Fuchs, 1996; Croxson, 1998). Over this period, the proponents of this new academic field – Anthony Culyer; Alan Enthoven; Rashi Fein; Martin Feldstein; Victor Fuchs; Michael Grossman; Herbert Klarman; Willard Manning; John Newhouse; Dorothy Rice; Frank Sloan; Kenneth Warner; Burton Weisbrod; and others – articulated a large body of economic expertise on health and health care. Working in public health schools, economics departments and government agencies, these economists also established research centres (for example, the National Bureau of Economics Research's (NBER) Health Economics Programme; the University of York's Centre for Health Economics) and research groups (for example RAND's Health Insurance Experiment; the UK-based Health Economists' Study Group), launched academic journals (for example, *Journal of Health Economics*; *Health Economics*), worked as consultants for governments and businesses and ran MSc and PhD programmes.

The making of this assemblage of expertise, institutions and activities was closely associated with one of the most important economic trends in the postwar industrialised nations of North America and Europe: the sharp increase of health expenditures in relation to the gross domestic product (GDP) (Colvin, 1985, chapter 6; Fuchs, 1996; Croxson, 1998; Fuchs, 2012a). In the USA, for example, where this trend was the most marked, health expenditures grew from about 4 per cent of GDP in 1945 to about 13 per cent in 1990 (Fuchs, 2012a). There were different reasons for this sharp increase in health expenditures. First, there was an important rise in the overall cost of health care due to changing patterns of medical practice, new technologies and third-party payments associated with the development of medical insurance (Gordon, 2003, chapter 1). Second, there was a growing amount of public money spent on social insurance schemes like the National Health Service in the United Kingdom and both Medicare and Medicaid in the USA, which were created as part of the dominant, postwar welfarist agenda in order extend health-care coverage to at least the most vulnerable in the population (Ashmore *et al*, 1989, chapter 1; Croxson, 1998; Gordon, 2003, chapter 1). Third, there was a rising number of private health-care plans that were set up, often by large companies for the benefit of their employees (Gordon, 2003, chapter 1; Fuchs, 2012b). The growing public anxiety and debates generated by the sharp increase in health expenditures could not fail to arouse the curiosity of economists and provided those of them willing to work in this area with a multitude of opportunities, from government positions to consultancies and research grants (Colvin, 1985, chapter 6; Porter, 1995, chapter 7; Croxson, 1998; Fourcade, 2009, pp. 109–112; Grossman, 2012; Warner, 2012).

The expertise developed by health economists brought new ways to think about, problematise and investigate health. Steeped in the neo-classical economic tradition predominant at the time, this expertise was free of any overriding political agenda, mostly applied and rather hybrid (Culyer *et al*, 1977; Fuchs, 1987; Morgan and Rutherford, 1998). Many of the economists who started working on health in the 1960s and 1970s came from the field of industrial organisation (for example, Klarman, 1965; Ginzberg and Ostow, 1969; Fuchs, 1972; Feldstein, 1979). They were interested in what they called ‘the health-care industry' – a service industry centred on physicians, hospitals and public health. Often with a view to improve efficiency, their research explored different aspects of this industry, including: the factors like income, price and tastes impacting on the demand for health care; the characteristics and supply of health manpower like doctors and nurses; and the organisation and functioning of production units like hospitals. Some of the first economists to work on health also came from the field of public policy economics (for example, Fein, 1958; Weisbrod, 1961; Rice, 1966; Feldstein, 1967). Escalating government expenditures in the postwar era, which were due to the state's increasing involvement in a number of areas from the construction of roads to the provision of health, rapidly led to questions about how best to allocate these growing public resources. Public policy economists were very successful in responding to this demand, offering policymakers ‘scientifically defensible' studies and procedures ‘to aid [them] make rational choices among alternative projects' (Weisbrod, 1961, p. xviii; cf. also: Porter, 1995, chapter 7; Fourcade, 2009, pp. 109–112). In the field of health, these included calculations of the costs of diseases to the economy and cost-benefit analyses of public health interventions. Yet another, smaller group of early health economists came from labour economics and human capital theory (for example, Mushkin, 1962; Grossman, 1972). Focusing on health rather than health care, their somewhat more theoretical work examined how individuals invested in their own health through a range of actions from purchasing medicines to eating well and how this investment was dependent on factors like age, education and income.

Interestingly, most of the economists working in these new areas of research were keen to emphasise that health and health care were ‘special' and did not always easily lend themselves to standard neo-classical economic approaches (Colvin, 1985, chapter 6). In other words, health and health care had unique characteristics that jarred with some of the concepts and models customarily used by economists and necessitated other kinds of methods and treatments. As Kenneth Arrow (1963, p. 948) explained in his seminal article on *Uncertainty and the Welfare Economics of Medical Care*: health has ‘special characteristics' that ‘distinguish it from the usual commodity of economics textbooks' and ‘establish a special place for [it] in economic analysis'. Perhaps the most remarkable issue for these economists was the way health and health care did not sit well with many of the assumptions that underlay the notion of the competitive market (for example, Arrow, 1963; Klarman, 1965; Feldstein, 1967; Culyer, 1971; Fuchs, 1972). To start with, the unpredictable incidence of illness meant that, in contrast to other commodities like food or clothing, the demand for health care was irregular and uncertain, making it difficult for patients to plan and calculate potential costs. Similarly, consumer rationality was deemed to be jeopardised by the fact that patients generally lacked the necessary information about their illnesses and had to rely on doctors for advice that should be completely divorced from any profit motive. Furthermore, individual choices about health were often held to generate positive or negative externalities for other members of society, as with decisions to purchase immunisations against infectious diseases. Most health economists thought that these uncertainties, information deficits and externalities caused market failures that had to be corrected through government interventions.

### Tobacco as an economic issue

From the 1970s onwards, as smoking was becoming a major public health issue in North America and Europe, some health economists started to apply the questions, concepts and methods that they had developed in relation to health and health care to tobacco (Berridge, 2007; Brandt, 2007). By the 1990s, these economists had produced an extensive body of knowledge on smoking and health, which soon became known as ‘the economics of tobacco' (Chaloupka and Warner, 2000). An important part of this knowledge was the studies that sought to determine the economic costs of smoking to the national economy (for example, Luce and Schweitzer, 1978; Leu and Schaub, 1984; Warner *et al*, 1999). Building on previous work done by health economists on the costs of diseases, these studies were often marred by controversies as to what costs should be included in the calculations. Another important part of tobacco economics, which is more relevant to us, was the analyses on the effectiveness of tobacco control measures like health campaigns, advertising bans and sin taxes – as excise taxes on cigarettes were often referred to (for example, Atkinson and Townsend, 1977; Warner, 1977; Lewit and Coate, 1982; Manning *et al*, 1989). Drawing on earlier health economics research on both the cost-effectiveness of health interventions and the demand for health, these analyses sought to determine how efficient these different tobacco control measures were by measuring their impact on the demand for cigarettes.

The first health economists to carry out extensive work on tobacco taxation were Joy Townsend at the British Medical Research Council, Kenneth Warner at the University of Michigan's School of Public Health, and Michael Grossman with some of his colleagues at the NBER Health Economics Programme like Eugene Lewit, Douglas Coate and, later on, Frank Chaloupka. Others joined from the late 1980s onwards, as efforts against smoking intensified and funding for research on tobacco control, like that from the Robert Wood Johnson Foundation in the USA, increased. They included: Willard Manning, Jeffrey Wasserman and others at RAND; Robert Leu at the University of Bern, Switzerland; Tei-Wei Hu at the University of California, Berkeley; and Markku Pekurinen at the Finnish National Public Health Institute. For the majority of these economists, the primary reason for working on taxation was to further develop and improve public health and tobacco control policy. Indeed, most of them shared the concerns of the anti-smoking movement and often actively supported it. Townsend, for example, who started working on taxation in the early 1970s, became increasingly active in the international tobacco control movement, regularly attending the World Conferences on Tobacco or Health and working as an expert for the WHO's first European *Action Plan on Tobacco* (for example, Atkinson and Townsend, 1977; Townsend, 1988; Townsend, 1996; cf. also Berridge, 2007, pp. 129–131). Similarly, Warner, who began writing on taxation in the early 1980s, was closely associated with anti-smoking efforts both in the USA and internationally (for example, Warner, 1984b; Warner, 1986; Warner, 1990; Warner, 2012).

However, for a small minority of these health economists the reasons for working on tobacco taxes were slightly different. The best example is probably Grossman, who carried out research on taxation from the late 1970s onwards, and Chaloupka, who began working on the topic as Grossman's PhD student in the late 1980s (for example, Lewit *et al*, 1981; Grossman, 1985; Chaloupka and Grossman, 1996; Chaloupka and Wechsler, 1997). While they were interested in the policy dimensions of their research and not unsympathetic to the concerns of the anti-smoking movement, they also saw their work as contributing to the economics of addiction (Chaloupka, 2012; Grossman, 2012). Specifically, they saw their work as feeding into a collaborative effort with Chicago School economist and neoliberal thinker Gary Becker – with whom Grossman had completed his doctoral thesis on human capital and health in the late 1960s – to develop and test his theoretical model of rational addiction (for example, Becker and Murphy, 1988; Chaloupka, 1991; Becker *et al*, 1994). For Becker, the consumption of addictive goods – defined as goods for which past consumption stimulates and reinforces current consumption – was a rational behaviour that could be explained by rational choice theory. Consequently, his model conceived the consumption of addictive goods like alcohol, tobacco and cocaine as a rational choice made by utility-maximising individuals on the basis of their income, prices and the risk of becoming dependent. This work on addiction was part of Becker's imperialist agenda to apply economic methodologies to issues commonly thought to be the domain of sociologists, psychologists and political scientists such as education, racial discrimination, crime, marriage and organ donation (Foucault, 2004; Medema, 2011). It was also related to Becker's conviction that the war on drugs had failed and his campaign for a new approach to drugs, namely: the legalisation of drugs combined with a high sin tax on consumption, restrictions on selling to minors, public education about the dangers of drugs and severe punishment for those driving or working while on drugs (Becker, 1997; Becker, 2001; Thornton, 2005).

By the late 1980s, research on tobacco taxation had started to coalesce into a coherent and stable body of work. A key finding was the ascertainment that price was a critical factor in reducing demand for tobacco products and that increasing taxes was one of the most powerful measures to combat smoking (Atkinson and Townsend, 1977; Lewit *et al*, 1981; Warner, 1986; Chaloupka and Grossman, 1996; Townsend, 1996). This research did not just determine the effectiveness of tobacco taxes but also examined in detail how they worked. To start with, it measured the price elasticity of tobacco products, estimating it to be around minus 0.5 (Lewit and Coate, 1982; Pekurinen, 1989; Townsend, 1993). This means ‘that, on average, cigarette consumption reduces about 0.5 per cent for every 1 per cent increase in its real price' (Townsend, 1996, p. 132). This research also eventually made clear that about half of the impact of price on cigarette consumption results in equal measure from its effect on smoking prevalence and smoking intensity (Chaloupka *et al*, 2012). Moreover, it showed that the impact of tax increases is more marked for young adults and for members of low socio-economic groups, for whom the effects of public information and education programmes are least effective (Lewit *et al*, 1981; Townsend, 1987). Furthermore, this research determined the type and rate of tax needed to decrease smoking prevalence by a given amount and drew attention to the fact that inflation will erode the impact of taxes unless these are increased accordingly (Townsend, 1993). Finally, it also made clear that a tax rise does not only improve a country's health but also its finances, as the tax rise largely compensates for the decrease in cigarette consumption (Warner, 1984b).

It is interesting to note that many health economists working on tobacco taxation were concerned with whether or not a government intervention like taxation was justifiable in economic terms. Not unlike the argument that had been made about health and health care, they claimed that the market failed to work efficiently in relation to tobacco and that governments had therefore to intervene through taxation and other public health measures (for example, Atkinson and Townsend, 1977; Lewit *et al*, 1981; Leu and Schaub, 1984; Manning *et al*, 1989; Pekurinen, 1992). Even a free market enthusiast like Gary Becker was happy to acknowledge market failure in relation to tobacco and to sanction a tax on tobacco products, which he saw as a ‘social tax' whose purpose was to correct the ‘social costs' associated with smoking (Becker, 1997, p. 150; cf. also: Becker, 2001; Becker, 2012). While most economists agreed that there was market failure in relation to smoking, they disagreed about the exact reasons for this failure. All, including Becker, concurred that one important reason was that, contrary to the tenets of neo-classical economic theory, consumers did not make informed choices in relation to smoking and did not bear all the costs of their choices. Indeed, as these economists pointed out: smokers were not fully aware of the high risks in terms of both health and addiction; and they imposed negative externalities onto non-smokers through passive smoking and running medical bills that were covered by social insurance schemes (Chaloupka, 2012; Warner, 2012). Many economists also argued that another reason for market failure was addiction itself, which made it impossible for smokers to take rational decisions – a point contested by researchers like Grossman, Chaloupka and Becker for whom the consumption of addictive goods was a rational behaviour (for example, Atkinson and Townsend, 1977; Leu and Schaub, 1984; Manning *et al*, 1989).

This knowledge on tobacco and taxes produced by economists helped to markedly transform the way taxation was perceived by the public health community. Public health experts had identified taxation as a possible smoking control measure in the 1970s already (Fletcher *et al*, 1971, p. 453; WHO, 1975, pp. 25 and 81–82; WHO, 1979, pp. 32 and 58–60; Roemer, 1982, pp. 73–75). But, until the 1990s, it remained extremely unpopular among the public health community who generally ignored it (Scott and Dickert, 1993; Warner, 2012). At best, public health experts considered taxation to be a minor and complementary measure that could be used to support and augment public information and education campaigns, which were the preferred tobacco control strategies at the time (WHO, 1975, pp. 23–24; WHO, 1979, pp. 45–53; Roemer, 1982, pp. 73–75). The reasons for this perception of taxation were multiple. First, taxation was tainted by its association with economists who were often viewed with suspicion and even hostility by public health activists (Fuchs, 1996; Croxson, 1998). Second, most public health experts did not really understand how taxation worked or whether it worked at all, with many believing that price had no impact on addicts (for example, Fletcher *et al*, 1971, p. 453; WHO, 1975, pp. 31–32; WHO, 1979, p. 59; Roemer, 1982, pp. 73–75). Third, many in the public health community were uneasy about the regressive nature of tobacco taxes, which meant that they hit the poor the hardest (Scott and Dickert, 1993; Chaloupka *et al*, 2012). Fourth, several public health advocates thought it was ‘reprehensible' to try and ‘change people's behaviour by using an extrinsic motivator like price rather than an intrinsic one like being concerned about one's health' (Warner, 2012). The research done by health economists was critical in transforming the perception of taxation among the public health community from a tobacco control policy that was unpopular and ignored to one that has become viewed as the most powerful measure to curb smoking (Scott and Dickert, 1993). As Kenneth Warner (2012), one of the first economists to work on tobacco taxes, makes clear:

There has been a complete change in the way the public health community perceives tobacco taxes … . In the early 1980s public health experts had an incredibly strong negative reaction towards taxes … . Now, taxes are viewed as the key tobacco control strategy … . The work of economists was critical in bringing this change.

## Tobacco and the Developing World

The second development that made it possible for taxation to become a critical tool of current international efforts to curb smoking in the global South was the problematisation of tobacco in developing countries. As this section shows, the realisation that smoking was becoming an important problem in the developing world led public health experts to suggest taxation as a possible solution and to call for economic research on taxation in the global South.

For a long time, tobacco was considered to be an issue that was exclusive to the rich, industrialised nations in the North (Brandt, 2007, chapter 9). Indeed, few public health experts imagined that tobacco could be a problem for developing countries. For them, developing countries were beset by infectious diseases, malnutrition and poverty; not NCDs and contributing risk factors like smoking. From the late 1970s onwards, this understanding progressively changed and tobacco became recognised as a problem for countries in the global South. An important moment in this process was the publication of Mike Muller's 1978 *Tobacco and the Third World* and Bo Wickerström's 1979 *Cigarette Marketing in the Third World*. Written by development experts, these two publications made it clear that a tobacco epidemic was on its way in the global South. They did so by showing how Western tobacco companies were hard at work creating new markets in Latin America, Asia and Africa through the setting up of efficient distribution systems, aggressive marketing strategies and intense lobbying of governing elites. They also showed how these companies were successfully pushing farmers in the developing world to cultivate tobacco in lieu of food crops. This, they argued, was causing increased poverty, higher mortality and morbidity, desertification and food shortages. Public health experts were quick to pick up on Muller and Wickerstrom's work and begin identifying smoking as a problem for developing countries (Ramström, 1985). A significant event in that respect was the publication in 1983 of *Smoking Control Strategies in Developing Countries*, a report in which the WHO recognised the rapid spread of a smoking epidemic in developing countries and suggested measures to halt its progress. During the next 15 years, the problem of smoking in the developing world would become increasingly acknowledged, studied and discussed in official reports, scholarly texts and at conferences (for example, Crofton, 1984; Chapman *et al*, 1986; Surgeon General, 1992; Bellagio Statement, 1995).

The way in which the problem of tobacco in the developing world was portrayed varied little throughout the 1980–1990s. First, as Muller and Wickerström had showed, the smoking epidemic in the global South was deemed to be due to ruthless transnational tobacco corporations searching for new markets (for example, WHO, 1983, pp. 8–9; Stebbins, 1990, p. 229; Mackay, 1991, p. 153). Second, the rise in smoking and smoking-related diseases in developing countries was understood to be part of a wider ‘epidemiological transition' taking place in those countries at that time: the increase in NCDs and their growing impact on mortality and morbidity rates (Jamison *et al*, 1984; Feachem *et al*, 1992; Jamison *et al*, 1993). Third, the increase of tobacco-related diseases in developing countries was thought to be particularly disastrous because of the additional medical and financial burden it created for these countries that, unlike the rich industrialised nations of the North, were still struggling with infectious diseases and malnutrition (for example, WHO, 1983, p. 9; Crofton, 1984, p. 270; Chapman *et al*, 1994, pp. 189–190). Fourth and finally, the rise of smoking and smoking-related diseases in the developing world was often seen as a harmful, unintended side effect of the industrialisation and modernisation process, which developing countries had to go through (for example, Warner, 1984a, p. 37; Stebbins, 1990, p. 228).

Although the way the problem of tobacco in the global South was portrayed varied little during the 1980–1990s, the manner in which it was measured became increasingly sophisticated. For most of the 1980s, the evidence for the growing numbers of smokers and smoking-related diseases in the developing world was ‘patchy': reports from doctors working in the field and a few small hospital and community surveys (Crofton, 1984, p. 269; Chapman *et al*, 1994, p. 189; Vateesatokit, 2003, p. 161). More rigorous and sophisticated evidence began appearing from the late 1980s. To start with, a growing number of developing countries started conducting regular national surveys to assess smoking rates. There was also a multiplication of epidemiological studies on smoking-related mortality and morbidity in the global South. Finally, there was the work of Richard Peto, Alan Lopez and their colleagues at the WHO that purported to produce credible estimates for worldwide smoking-related morbidity and mortality (for example, Peto *et al*, 1994; Peto *et al*, 1996). This increasingly sophisticated epidemiological evidence certainly strengthen public health experts' conviction that tobacco had become a problem for the global South.

The realisation that smoking was becoming an important problem in the developing world led public health experts to suggest possible solutions. These solutions included: a whole array of policies and measures to combat the epidemic (for example, Roemer, 1982; WHO, 1983; World Bank, 1993b); workshops run by organisations like the International Union against Cancer to build up tobacco control advocacy networks in developing countries (Wood, 1994); and calls for research to understand the specificities of the smoking epidemic in the global South (for example, Surgeon General, 1992, p. 10; Bellagio Statement, 1995, p. 1110). Importantly for us, taxation was one of the policies and measures suggested by public health experts to tackle the smoking epidemic in the developing world. This is not all that surprising given that these policies and measures were generally modelled on those in existence in the rich, industrialised nations of the North (Brown and Bell, 2008). Nor is it surprising that, mirroring what had happened in the North, the understanding of tobacco taxation in the global South shifted from a measure that was unpopular and minor at best (for example, Roemer, 1982, pp. 73–75; WHO, 1983, pp. 56–58) to one that was essential (for example, Surgeon General, 1992, pp. 127–136; Roemer, 1993, chapter 8; World Bank, 1993b, p. 2; Chapman *et al*, 1994, p. 191). Understandably, this shift was accompanied by calls for research on the specificities of the economics of tobacco and taxation in the global South (for example, Warner, 1990; Surgeon General, 1992, p. 10).

## The World Bank and Tobacco Taxes

The third development that was critical in making taxation into an essential tool of current international anti-smoking efforts in the developing world was the active promotion of the Western economic knowledge on tobacco taxation by World Bank economists and epidemiologists from the late 1990s onwards. While the problematisation of tobacco in the developing world had led to taxation being suggested as a possible solution, this active promotion ensured that taxation was effectively disseminated to and used by countries in the global South.

One of the reasons for the Bank's critical role in promoting tobacco taxation in the global South has been its increasing involvement with international health over the last few decades. The Bank's initial engagement with health was driven by a change in thinking about development in the 1970s (Finnemore, 1997; Fair, 2008). Both at the Bank and within the wider development community, there was a growing recognition that development should be about poverty alleviation rather than just economic growth. This meant that the Bank had to complement its usual development strategies aimed at improving countries' physical capital (roads, railways, agriculture and so on) with strategies aimed at improving their human capital like education and health. This newfound interest in health led the Bank to create a new department – the Health, Nutrition and Population department (HNP) – and offer loans to countries for health-related projects (for example, family planning, nutrition programmes and so on). The Bank's involvement in international health steadily increased during the 1980–1990s, so much so that at the end of the century it was lending countries over 2 billion dollars for health-related projects and had become the world's premier health institution, pushing the WHO to the sidelines (Abbasi, 1999). An important part of the Bank's work on health during that time was concerned with the epidemiological transition then taking place in developing countries and how to ease the additional financial burden this generated for these countries' health systems. The solutions put forward by HNP epidemiologists and economists like Dean Jamison, Anthony Measham and Richard Feachem were varied (for example, Jamison *et al*, 1993; World Bank, 1993a). Some involved focusing efforts on high-morbidity and mortality diseases as well as privileging the most cost-effective health interventions. Others, more controversially and no doubt influenced by the neo-liberal critique of development economics that was then becoming predominant, included structural adjustment policies like public deficit reduction and privatisation strategies (Rowden, 2009).

It is as part of this increasing engagement with health that, in the late 1980s, the Bank first got involved with tobacco control. Its involvement was twofold. The first instance was the Bank's revision of its lending policy for tobacco production. The Bank had long lent money to countries to develop their tobacco production infrastructure; indeed, as with any other industry, investment in the tobacco industry was deemed to generate economic growth and development. With the recognition of tobacco and NCDs as a problem for developing countries in the 1980s, the Bank's tobacco lending policies became increasingly criticised. As a result, following efforts from HNP epidemiologists and economists like Howard Barnum, Anthony Measham and Ann Hamilton, the Bank stopped lending for tobacco production in 1991 (World Bank, 1992; World Bank, 1993b). The second instance was the use of tobacco and tobacco control policies by HNP experts in the first edition of *Disease Control Priorities in Developing Countries* (1993). A key conclusion of this publication was that, given the growing costs associated with NCDs in the global South, governments in developing countries should concentrate their efforts on cost-effective measures targeting diseases affecting the largest number of their citizens. Tobacco control policies were deemed to be the perfect illustration of such measures by Dean Jamison and his colleagues; indeed, they were extremely cost-effective and tackled one of the future's biggest causes of mortality and morbidity in the developing world (Jamison *et al*, 1993, chapters 1, 2, 21 and 29 and annex A).

Given the Bank's interest and work not only in economics and development but also in health and tobacco control, it is not surprising that it actively promoted and disseminated existing economic knowledge on tobacco taxation to developing countries between 1995 and 2005. An important component of the Bank's promotion and dissemination efforts was the publication of both *Curbing the Epidemic* and *Tobacco Control in Developing Countries* (Jha and Chaloupka, 2000). These two publications, which outlined and discussed the relevance of tobacco economics for the developing world, were written by a team led by Prabhat Jha, a young and dynamic HNP epidemiologist, and composed of renowned economists and public health experts, including: Frank Chaloupka, Tei-Wei Hu, Dean Jamison, Judith Mackay, Markku Pekurinen, Richard Peto, Ruth Roemer, Joy Townsend, Kenneth Warner and Derek Yach. These two publications did three things in particular. First, they presented this knowledge in a form that was easily accessible and comprehensible to non-economists (World Bank, 1999, pp. 37–45; Jha and Chaloupka, 2000, chapter 10). Second, they showed the relevance of tobacco taxes for developing countries by explaining that it was the most cost-effective measure to address the tobacco-related epidemic of chronic diseases and premature death that was now rapidly affecting the developing world (World Bank, 1999, chapter 1; Jha and Chaloupka, 2000, chapters 2–4). Third, they also justified tobacco taxes in economic terms, by explaining that, in the case of tobacco, there were market failures that necessitated governmental interventions, including taxation (World Bank, 1999, chapter 3; Jha and Chaloupka, 2000, chapter 7).

The Bank's efforts to promote and disseminate knowledge on tobacco taxation did not stop with the release of these two publications but continued until 2005 through a multiplicity of activities organised by HNP economists Aida Yurekli and Joy de Beyer (World Bank, 2005). First, the Bank sent representatives to the FCTC negotiations to comment on economic issues including taxation. Second, the Bank distributed over 17 000 copies of *Curbing the Epidemic* in over 20 languages and ran a website dedicated to tobacco economics. Third, the Bank conducted over 60 seminars and workshops on the economics of tobacco in general and taxation in particular, which were held in developing countries and open to governmental officials, public health experts and tobacco control advocates. Fourth, the Bank drafted and published a manual on how to conduct economics analysis on tobacco control in developing country – the *World Bank Economics of Tobacco Toolkit* – with one whole chapter dedicated to taxation (Yurekli and de Beyer, 2004, Tool 4). Fifth and finally, the Bank also commissioned research papers and notes on the economics of tobacco and taxation in over 25 developing countries.

The Bank's efforts had a remarkable effect. In the decade that preceded the publication of *Curbing the Epidemic* in 1999, there had been only three research projects on the impact of price on tobacco consumption and one attempt to use tobacco taxation as a public health tool in developing countries: Simon Chapman and his colleagues' research on the effects of taxation on cigarette consumption in Papua New Guinea in the late 1980s (Warner, 1990); Supakorn Buasai and his colleagues' successful attempt to lobby the Thai government to increase taxes on cigarettes to reduce smoking prevalence in the mid-1990s (Vateesatokit, 2003, pp. 162–163); Zhengzhong Mao and J.L Xiang's work on the relation between price and smoking prevalence in China in the late 1990s (Mao and Xiang, 1997; Mao *et al*, 1997); and Iraj Abedian's Economics of Tobacco Control in South Africa project funded by the IDRC in the late 1990s (IDRC, 2012). In contrast, in the decade that followed, there have been countless initiatives, funding schemes and research projects on tobacco tax policies in the global South, as outlined in great detail in the first section of this article.

## The Economisation of Global Health beyond Structural Adjustment

There has been a growing critique of the often superficial and un-reflexive way in which social scientists have depicted neo-liberalism as a harmful force at the heart of most of the political and economic transformations that have taken place in the last few decades (for example, Clarke, 2008; Collier, 2009; Ferguson, 2009; Mirowski and Plehwe, 2009; Collier, 2011). These authors have pointed out that the omnipresence and omnipotence attributed to neo-liberalism in these depictions threatens to deprive the concept of any analytical value. Indeed, it makes little sense to say that everything – from knowledge and forms of governance to subjectivities and spaces – is neoliberal or shaped by neo-liberalism. Similarly, they have argued that these depictions frequently misunderstand neoliberal thought. In particular, they have showed that: neo-liberalism is often mistakenly equated with other related yet distinct bodies of knowledge such as neo-classical economics; neo-liberalism is repeatedly portrayed in ways that are too monolithic and ignore the plurality of thought among and tensions between the many factions that make up the transnational networks of intellectuals and think tanks centred around the Mont Pèlerin Society; and neo-liberalism is regularly caricatured as when deemed to lack any notion of justice or the social. Likewise, these authors have contended that most depictions of neo-liberal ideas and practices tend to overlook their instability and the possibility of them being re-appropriated and transformed over time and space. Finally, they have also deplored the unexamined belief informing much social science research that neo-liberalism is necessarily negative and harmful. The genealogy of tobacco taxes outlined above contributes to this critique by highlighting some of the weaknesses of the standard story about the economisation of global health found in the social sciences literature.

### The standard story

As already alluded to, the usual story about economics and global health equates the economisation of global health with the structural adjustment policies that have been predominant in international development over the past 30 years (for example, Whitehead *et al*, 2001; Thomas and Weber, 2004; Maciocco, 2008; Rowden, 2009; Schrecker *et al*, 2010).^1^ Often referred to as ‘the Washington Consensus', structural adjustment policies are a set of policies aimed at stabilisation, liberalisation and privatisation (Mosley *et al*, 1991, chapter 1; Williamson, 2004; Rowden, 2009, chapters 6–8; Plehwe, 2009a). They comprise: currency devaluation; inflation control policies; public deficit reduction strategies; the removal of trade barriers such as tariffs and quotas; the opening of financial markets to foreign investments; the abolition of price controls; the deregulation of key industries through the elimination of licensing regimes and other market entry impediments; the softening of labour regulations on minimum wages, working hours and redundancy; and the privatisation of state-owned enterprises. These policies began to dominate the field of international development in the early 1980s. Indeed, it was then that they became a standard lending modality of general budgetary support loans from the International Monetary Fund and the World Bank, thus forcing developing countries to roll back the state and introduce free market reforms in exchange for financial support (Mosley *et al*, 1991, chapter 2; Rowden, 2009, chapter 6; Collier, 2011, chapter 6).

As the usual story on the economisation of health rightly claims, structural adjustment policies are closely related with neo-liberalism and, more specifically, with the neoliberal critique of state-centred development economics, which Toye (1987) has called the ‘counter-revolution in development economics' (Mosley *et al*, 1991, chapter 1; Plehwe, 2009b; Collier, 2011, chapter 6). In the three decades that followed World War II, the field of international development was dominated by a statist model. Promoted by economists like Gunnar Myrdal, Raul Prebisch and Walt Rostow, this model aimed to modernise poor countries through an accelerated, state-led industrialisation process based on strategies such as: central planning, infrastructure development, selective subsidies, public ownership, price controls, trade tariffs and labour regulations. From the 1950s onwards, scholars associated with the Mont Pèlerin Society and other neoliberal think tanks like Peter Blau, Herbert Frankel and Deepak Lal began to articulate a critique of this state-led model (Toye, 2009; Plehwe, 2009b; Collier, 2011, chapter 6). Most generally, they argued that the very division of the world into a developed and underdeveloped area, with the latter requiring special economic development measures, was fallacious as the market mechanism was supposed to be the same everywhere (Strassmann, 1976). They also criticised the infatuation with industrialisation, claiming that it discriminated against poor farmers in rural settings, and lamented the pessimism about the promise of international trade to generate growth. More importantly for us perhaps, these neoliberal critiques had a ‘profound scepticism toward the state, frequently regarded as a self-interested bureaucracy inclined towards predatory behaviour', and contended that ‘government interventions in market processes encouraged rent-seeking dynamics' and other distortions that were ‘inimical to productive economic activity and genuine development' (Bair, 2009, p. 364). To deliver genuine development, they argued, one needed to correct these distortions by ‘opening up economic activity to the free play of market forces' (Mosley *et al*, 1991, p. 24). In particular, one needed to roll back the state and embrace both domestic entrepreneurship and international trade as alternative engines of growth. By the late 1970s, this neoliberal critique had become the dominant paradigm and was instrumental in shaping structural adjustment policies from the 1980s onwards.

These structural adjustment policies, as the standard story on the economisation of global health further explains, have had a hugely detrimental effect on health in the global South (for example, Whitehead *et al*, 2001; Stuckler *et al*, 2008; Rowden, 2009; Stuckler *et al*, 2009; Schrecker *et al*, 2010). There is little doubt that structural adjustment policies have had a major impact on the way medical care and public health is organised, managed and financed in the developing world (for example, World Bank, 1987; World Bank, 1993a). To start with, public deficit reduction strategies have brought about: salary caps and lay-offs for doctors and nurses working in the public sector, often causing them to emigrate in search of better working conditions; the closure of public hospitals and health-care services; the elimination of public subsidies for any non-essential public health programmes, medical services and drugs; and the development of alternative sources of revenue through the establishment of user fees and private insurance schemes (Stuckler *et al*, 2008; Rowden, 2009, chapters 9–10). At the same time, privatisation schemes have led to an increasing number of hospitals and health-care centres being managed along market principles by private companies and public–private partnerships, thereby making access to health care difficult for many (Rowden, 2009, chapters 9–10). Likewise, deregulation policies in the pharmaceutical market have led to an ‘irrational use of medicines' caused by the increased ‘sale of drugs without prescription by unqualified people who have financial incentives to overprescribe' (Whitehead *et al*, 2001, p. 834). Unsurprisingly, these impacts on the organisation, management and financing of health care brought about by structural adjustment policies have led to a rise in both morbidity and mortality rates, especially in the countries of the ex-Soviet Union and sub-Saharan Africa (Whitehead *et al*, 2001; Stuckler *et al*, 2009; Schrecker *et al*, 2010).^2^

### An alternative narrative on economics and global health

The genealogy of tobacco taxes presented in this article does not contest that neo-liberal structural adjustment policies have been influential in and often detrimental to international health over the past 30 years. Rather, it challenges some of the limitations inherent to the standard story outlined above and suggests that it is not the only narrative about economics and global health that can and should be told.

First of all, this genealogy shows that one cannot reduce the economisation of global health to neoliberal structural adjustment policies, as the standard story tends to do. Indeed, tobacco taxes are not a structural adjustment policy aimed at liberalisation, stabilisation and privatisation, but a public health policy aimed at reducing tobacco consumption and the growing burden of NCDs associated with it, on a par with indoor smoking bans and anti-smoking health education. Furthermore, tobacco taxes have a different genealogy than structural adjustment policies. In particular, they have not been made possible by the neoliberal counter-revolution in development economics, but by the emergence of health economics. These are two different projects developed by different people. Articulated by thinkers associated with the networks centred about the Mont Pèlerin Society, the neoliberal critique of development economics was a political project that sought to transform international development by rolling back the state and embracing free trade and markets. In contrast, health economics was a hybrid, mostly applied field of research free of any overriding political agenda where academic economists coming from industrial organisation, public policy and labour economics sought to empirically explore health-related issues like the organisation of the health-care industry, the cost-effectiveness of public health interventions and the demand function for health. Importantly, the genealogy of tobacco taxes outlined in this article shows that, while certainly not unheard of, the desire to roll back the state and the enthusiasm for markets that characterised the neoliberal counter-revolution in development economics had little resonance within health economics.^3^ If anything, most health economists thought that health was characterised by uncertainties, information failures and externalities that did not necessarily sit well with the market model and often had to be corrected through government action (Colvin, 1985, chapter 6). This way of thinking was even more marked for tobacco, with virtually all health economists concurring that there was market failure in relation to smoking and a need for government interventions like tobacco taxes (Jha and Chaloupka, 2000, chapter 7).

It is important to note that the fact that tobacco taxes are not related to the neoliberal critique of development economics does not mean that there are no connections between tobacco economics and other, different strands of the neoliberal tradition. As the genealogy of tobacco taxation presented here shows, the most significant connection between tobacco economics and neoliberalism was the one involving Gary Becker. A professor of economics at Chicago and an important neoliberal thinker, Becker was not himself a health economist and did not work directly on tobacco (Plehwe, 2009a; Medema, 2011). But, through his association with Michael Grossman and Frank Chaloupka, his theory of rational addiction and his imperialist agenda to apply economic approaches to issues traditionally outside economics had some influence on tobacco economics (Grossman, 2012). But, this influence should not be exaggerated and does certainly not warrant calling tobacco economics ‘neoliberal' in any meaningful sense. Most tobacco economists had no links with Becker and only very limited interest in his theory of rational addiction; often actively involved in the anti-smoking movement, their prime concern was to help improve tobacco control policy through the use of standard, neo-classical economic tools (Warner, 2012). Besides, Becker's work on addiction and, more broadly, on the legalisation of drugs had very little to do with the free market and anti-statist stance commonly associated with neoliberalism. Indeed, Becker admitted market failure in relation to tobacco, alcohol and drugs and called for government interventions like sin taxes, something other neoliberal thinkers explicitly disagreed with (Becker, 1997; Thornton, 2005; Becker, 2012).

It is also important to note that the fact that tobacco taxes are not related to the neoliberal critique of development economics and its free market agenda does not signify that they are somehow against or outside the market. Unlike prohibition or state monopolies, taxation is not an alternative to the market in terms of how to organise the production and exchange of tobacco (Thornton, 2005; Becker, 2012; cf. also: Davies, 2013). Tobacco taxes do not abolish the market by forbidding all production and exchange of tobacco products, as prohibition would do. Nor do tobacco taxes eliminate the market by allowing one single, state-owned company to control the entire national production, distribution and sale of tobacco products, as a state monopoly would do. Instead, taxation works in combination with the market. Indeed, tobacco taxes assume the existence of a functioning market in tobacco products. But, because of some failures, this market is not working as well as it could. Tobacco taxes are there to help correct these failures and thus allow the market to perform more efficiently. As Chaloupka (2012) explains:

The idea is that there is a market that is working at some level … but there are market failures that keep it from working as efficiently as it could … . Tobacco control measures like taxes are going to deal with those market failures.

Another weakness of the standard story about the economisation of global health is how it only focuses on the impact of structural adjustment policies on health and, consequently, the way it portrays the relation between economics and global health as unidirectional and harmful to health. As the genealogy of tobacco taxes outlined here shows, the relation between economics and global health is much more complex than that. Like the standard story, this genealogy demonstrates how influential economics can be for global health by describing the way health economists have made price and taxation so critical to the fight against smoking and thus transformed global tobacco control. But, the genealogy of tobacco taxes also offers many illustrations of how changes in health can influence economics. One of these is the way the continuing and marked increase in health expenditures in the rich, industrialised countries of the North led to the development of the field of health economics in the 1960s. Another illustration is how a shift in the epidemiology of disease – the realisation that tobacco-related diseases and, more generally, NCDs have become a key health issue for the developing world – made it possible for the economic expertise on tobacco taxation to be exported to the global South. Moreover, the genealogy of tobacco taxes outlined in this article also illustrates that economics is not only detrimental to health, as in the case of structural adjustment policies, but also beneficial to it. Indeed, as health economists have demonstrated, an economic strategy like tobacco taxation has an extremely positive impact on health: first, by markedly bringing down smoking prevalence and intensity; and, second, by generating increased revenues that can be reinvested in public health campaigns and other measures (Warner, 1984b; Townsend, 1996; Chaloupka and Warner, 2000).

## Conclusion

The present article explored the recent and extensive economisation of global health through a close analysis of one economic strategy – taxation – that has become central to international efforts to curb smoking in developing countries. Adopting a genealogical approach, it argued that three developments in particular made it possible for taxation to become so critical to global tobacco control today. The first of these developments was the articulation, by health economists in North America and Europe in the 1970–1990s, of a body of knowledge that transformed public health thinking about smoking by showing the marked impact of price on the demand for tobacco products. The second development was the problematisation of smoking in the global South from the late 1970s onwards, which made it possible for public health experts to suggest taxation as a solution to curb the tobacco epidemic in developing countries. The third development was the active promotion of tobacco taxes by World Bank epidemiologists and economists at the turn of the twenty-first century that allowed this strategy to be effectively used in the developing world.

As the article also showed, this genealogy does not sit comfortably with the standard story on economics and global health found in the social science literature (for example, Thomas and Weber, 2004; Stuckler *et al*, 2008; Rowden, 2009). This story reduces the economisation of global health to the structural adjustment policies aimed at stabilisation, liberalisation and privatisation that have been dominant in international development over the past 30 years. It also argues that these policies, which were the product of the neoliberal counter-revolution in development economics, have been extremely detrimental to health in the global South. The genealogy of tobacco taxation presented in this article did not dispute that neo-liberal structural adjustment policies have played a significant and damaging role in international health over the past 30 years. But, it challenged some of the limitations inherent to the standard story about economics and global health. First, it showed that the economisation of global health is not only about the neoliberal critique of development economics and structural adjustment policies but also about health economics, sin taxes and market failures. Second, by stressing how changes in health such as the global South's epidemiological transition have influenced economics and by emphasising the beneficial impact of taxation for health, it also showed that the relation between economics and global health is not necessarily unidirectional and detrimental to the latter. In doing so, this article contributed to the recent body of work that warns against the inflationary and often unthinking use of neo-liberalism to explain change (for example, Ferguson, 2009; Collier, 2011). More broadly perhaps, it calls for researchers to go beyond neoliberal structural adjustment policies and start telling other, different stories about the economisation of global public health.

## Footnotes

1 The attraction for these neoliberal policies has recently begun to wane (Boseley, 2012; Elliott, 2012; Lal, 2012).

2 However, see the recent critique by Gerry (2012), who argues that the statistical method used to correlate mass privatisation and higher mortality rates in the ex-Soviet Union is flawed.

3 Of course, some health economists did advocate market solutions in relation to the production and allocation of health. The work of Michael Cooper and Anthony Culyer, where they argued in favour of using the price mechanism to organise the supply of blood for transfusions and which they carried out in the 1960s with the support of the neoliberal Institute of Economic Affairs, is perhaps the best example (Fontaine, 2002). But that was a minority position as Cooper and Cuyler themselves recognised in their 1973 textbook on *Health Economics,* in which they explained that the ‘market-versus-state debate' was now ‘in the past' and that ‘the trend' in health economics was ‘towards empiricism, applying theory to solve real-world problems rather than engaging in abstract speculation' (1973, p. 7).
